# A 10-year bibliometric analysis of osteosarcoma and cure from 2010 to 2019

**DOI:** 10.1186/s12885-021-07818-4

**Published:** 2021-02-04

**Authors:** Wacili Da, Zhengbo Tao, Yan Meng, Kaicheng Wen, Siming Zhou, Keda Yang, Lin Tao

**Affiliations:** Department of Orthopedics, The First Hospital of China Medical University, 155 Nan Jing North Street, Shenyang, 110001 Liaoning China

**Keywords:** Osteosarcoma, Cure, Bibliometric analysis, Hotspots, Co-word biclustering analysis

## Abstract

**Background:**

In recent decades, the 5-year survival rate of osteosarcoma remains poor, despite the variety of operations, and exploration of drug therapy has become the key to improvement. This study investigates the contribution of different aspects in osteosarcoma and cure, and predicts research hotspots to benefit future clinical outcomes.

**Methods:**

The Web of Science and PubMed databases were queried to collect all relevant publications related to osteosarcoma and cure from 2009 to 2019. These data were imported into CiteSpace and the Online Analysis Platform of Literature Metrology for bibliometric analysis. Bi-clustering was performed on Bibliographic Item co-occurrence Matrix Builder (BICOMB) and gCLUTO to identify hotspots. Additionally, completed clinical trials on osteosarcoma with results past phase II were collated.

**Results:**

A total of 2258 publications were identified in osteosarcoma and cure from 2009 to 2019. China has the largest number of publications (38.49%), followed by the United States (23.03%) with the greatest impact (centrality = 0.44). The centrality of most institutions is < 0.1, and Central South University and Texas MD Anderson Cancer Center possess the highest average citation rates of 3.25 and 2.87. *BMC cancer* has the highest average citation rate of 3.26 in 772 journals. Four authors (Picci P, Gorlick R, Bielack SS and Bacci G) made the best contributions. We also identified eight hotspots and collected 41 clinical trials related to drug research on osteosarcoma.

**Conclusions:**

The urgent need exists to strengthen global academic exchanges. Overcoming multidrug resistance in osteosarcoma is the focus of past, present and future investigations. Transformation of the metastasis pattern, microenvironment genetics mechanism, alternative methods of systemic chemotherapy and exploration of traditional Chinese medicine is expected to contribute to a new upsurge of research.

## Background

Osteosarcoma, a common primary bone malignancy in children and adolescents, occurs primarily in the metaphysis of long bones and is characterized by early lung metastasis, high mortality and poor prognosis [1]. Limited by primitive medical care, amputation was the mainstream osteosarcoma treatment, with a 5-year survival rate of approximately 20%, and the defective appearance and function of the affected limb has a serious impact on patient psychology [2]. With the rapid development of medical science and technology, limb salvage in osteosarcoma has been adopted at a rate of more than 80% in the clinic and has gradually replaced the majority of amputation cases, but the survival rate is still poor [3]. This outcome might be related to many unsolved issues, such as complex pathogenesis, lack of novel adjuvant drugs and an imperfect evaluation system. Currently, the widely accepted strategy for osteosarcoma is surgery combined with neoadjuvant chemotherapy, which has a great capacity to shrink tumours and eliminate small lesions to ensure complete surgical resection and reduce tumour recurrence and metastasis [4]. Since the surgical method is generally determined, the key to improving the survival rate is drug therapy, which is also a difficulty and a hot issue for global orthopaedic and oncology experts.

Bibliometric analysis has become the best tool for exploring the detailed research trends in a certain field over time [5]. This analysis objectively presents the research contributions of different countries, institutions, journals and authors in scientific fields via qualitative and quantitative analysis and forecasts the research trends or hotspots. At the same time, it is worth explaining that the hotspots refer to the problems in a specific filed that has not been solved and is highly concerned by global scholars, or the research direction that needs to be broken through urgently and of great significance in the future. In addition, bibliometric analysis has also played a momentous role in formulating policy and clinical guidelines on various diseases [6]. However, no current bibliometric analysis has been conducted on osteosarcoma and cure, and even less attention has been focused on prediction of research hotspots. Our previous publications demonstrated that bi-clustering analysis has great advantages in exploring key areas of research and the related representative literature and can also be applied to verify hotspots [7, 8]. This study aims to conduct a comprehensive investigation of the current academic status and clinical issues in osteosarcoma, with a focus on cure and chemoresistance, and to predict the potential progress in this field over the next decades. We also summarize the representative clinical trials of drug therapy for osteosarcoma.

## Methods

### Data sources and collection

In recent years, the Science Citation Index Expanded and the Social Science Citation Index from Thomson Reuters Web of Science have been developed as the most authoritative and widely used bibliometric analysis databases. We performed a comprehensive collection of all of the original articles and reviews from the Web of Science from 2009 to 2019 with the following retrieval strategies: osteosarcoma AND (drug OR medicine OR medication OR remedy OR chemical OR cure) AND Language = English. Medical Subject Headings (MeSH) terms are a type of standard vocabulary that can be adopted to perform continuous co-word cluster analysis and reflect the main thrust of the literature [9]. We also conducted a similar online search in PubMed based on the screening criteria of (“Osteosarcoma”[Mesh]) AND (“drug” OR “medicine” OR “medication” OR “remedy” OR “chemical” OR “cure”), which was developed by the National Center for Biotechnology Information (NCBI) of the National Library of Medicine (NLM). No language restrictions were applied for all literature search and downloading processes, which were completed within one day on February 26, 2020 to avoid errors caused by frequent database updates. Clinical trials that had completed Phase II were obtained from ClinicalTrials.gov (https://clinicaltrials.gov/).

All data were independently collected by two authors (WCLD and SMZ) with an agreement rate that eventually reached 0.90 that implies a high degree of accordance [10]. The data obtained from Web of Science were converted to txt format and imported into CiteSpace V5.5.R1 SE, 64-bit (Drexel University, Philadelphia, PA, USA) and the Online Analysis Platform of Literature Metrology (http://bibliometric.com/) for subsequent bibliometric analysis. The data downloaded from PubMed were uploaded to Bibliographic Item co-occurrence Matrix Builder (BICOMB), a tool for hotspot analysis [11].

### Statistical methods

First, we analysed and summarized all kinds of indicators, including countries, institutions, journals, authors, H index and impact factor (IF) of the version (2019) of Journal Citation Reports (JCR) for all publications. Moreover, the annual publication quantities and growth trends of different countries/regions were found through the online bibliometric platform. CiteSpace was used to identify the collaborations among countries, institutions and authors. A co-occurrence analysis of the keywords was performed to predict research frontiers and new trends. The method of “time slicing” was also performed in CiteSpace, where we were free to set the years and the number of summarized papers for each slice. As for our analysis strategy was extracting the first top 50 papers in a year slice into a single network [7]. Depending on the purpose of our analysis, we chose different nodes for which the size represents the citation count or the quantity of publications [12, 13].

We conducted bi-clustering analysis on the selected publications and major MeSH terms/MeSH subheadings to explore the hotspots of osteosarcoma and cure. We also constructed a binary matrix with the original documents as columns and the major MeSH terms/MeSH subheadings as rows for further clustering through BICOMB and gCLUTO version 1.0, Graphical Clustering Toolkit (http://glaros.dtc.umn.edu/gkhome/cluto/gcluto/download), and the repeated bisection were applied to clustering analysis based on following parameters: I2 and cosine are selected as criterion functions and similarity functions respectively [7]. Moreover, the clustering with different number of clusters was redirected to ensure the optimal result of matrix visualization. The existing semantic relationships between major MeSH terms/MeSH subheadings and the source literature in the clusters were demonstrated through matrix and mountain visualization.

## Results

### Publishing trend

**Fig. 1:**
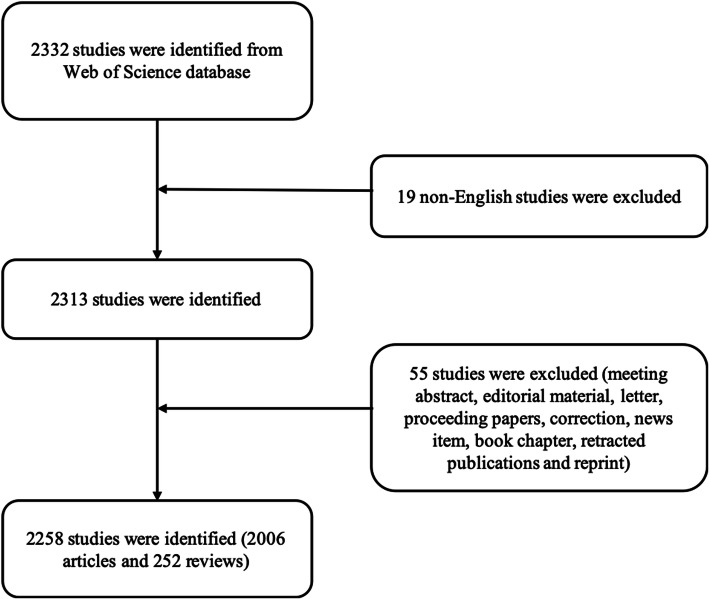
Flow diagram of the inclusion process

As shown in Fig. 1, 2258 publications (2006 articles and 252 reviews) met our inclusion criteria from 2009 to 2019. The publications related to osteosarcoma and cure continued to increase from 2009 to 2018, but a slight decline occurred in 2019, which may be due to the fact that osteosarcoma has reached a bottleneck period that the prognosis has not improved significantly, and the specific research directions of osteosarcoma was quite unclear which highlights the need for this study to objectively demonstrate the status quo and provide promising direction for future research (overall increase from 106 in 2009 to 303 in 2019, Fig. 2).

**Fig. 2:**
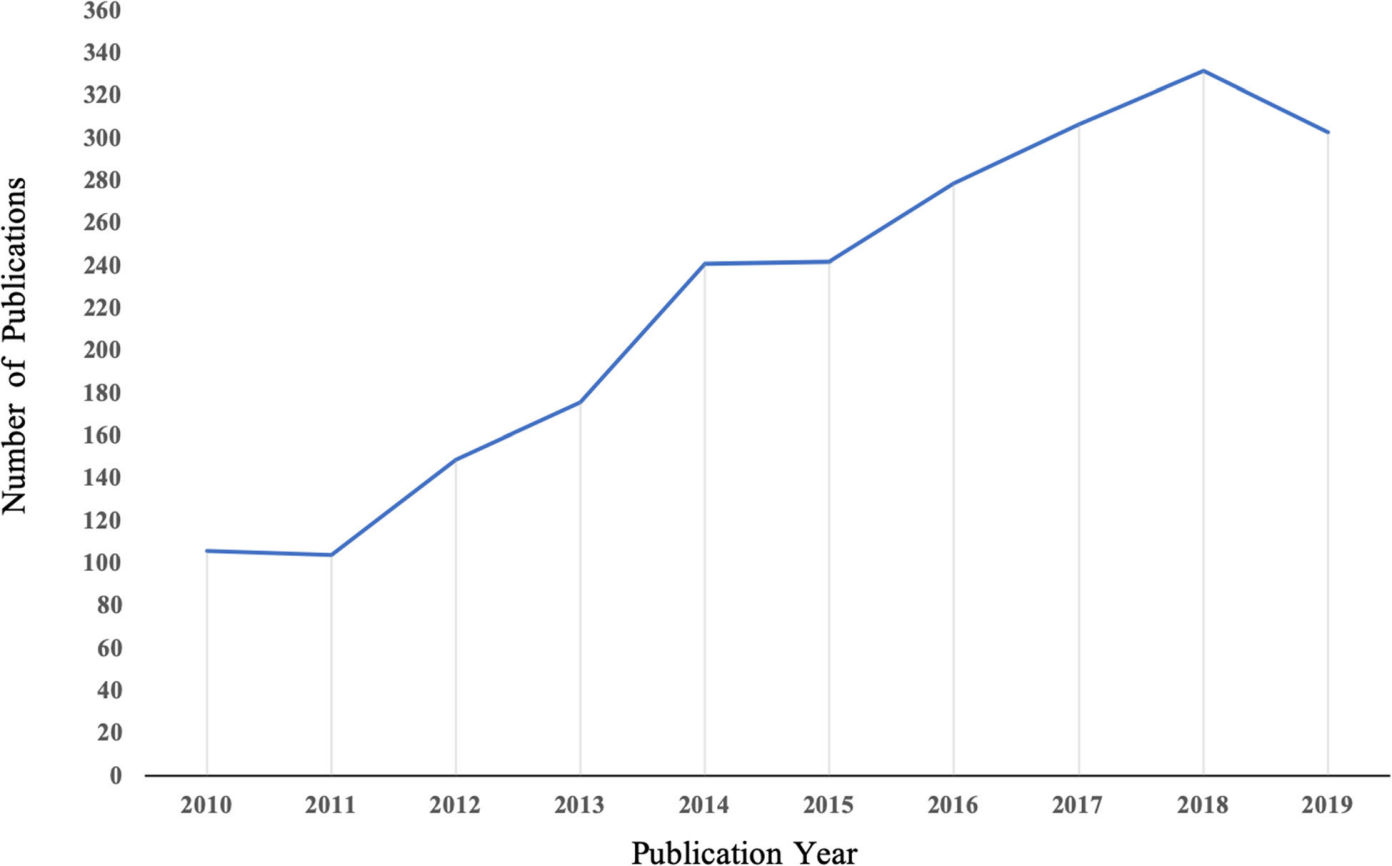
Growth trends of publications on osteosarcoma and cure from 2009 to 2019

### Contributions of countries and institutions to global publications

**Fig. 3:**
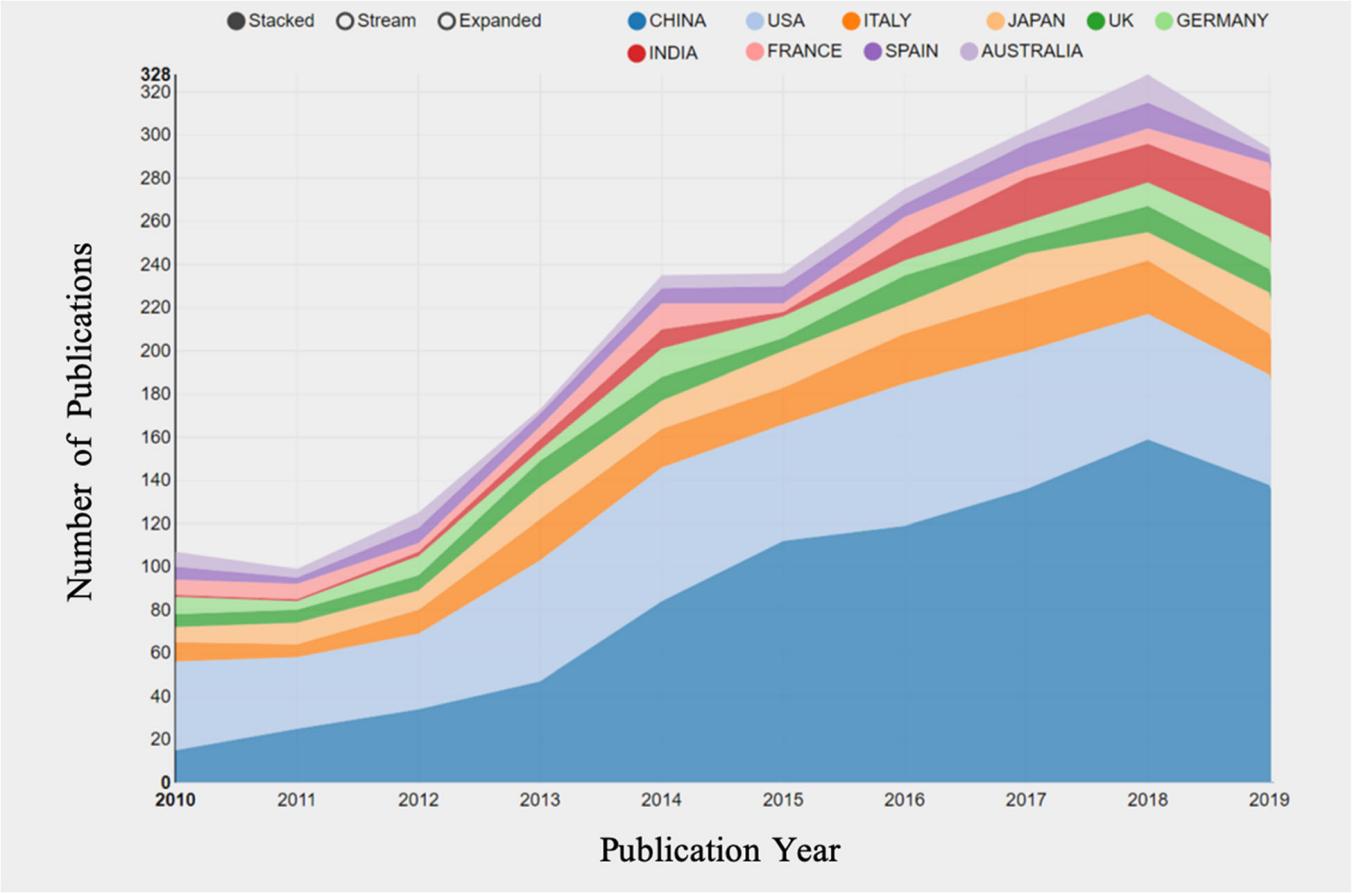
Growth trends of the top 10 countries on osteosarcoma and cure from 2009 to 2019

**Fig. 4:**
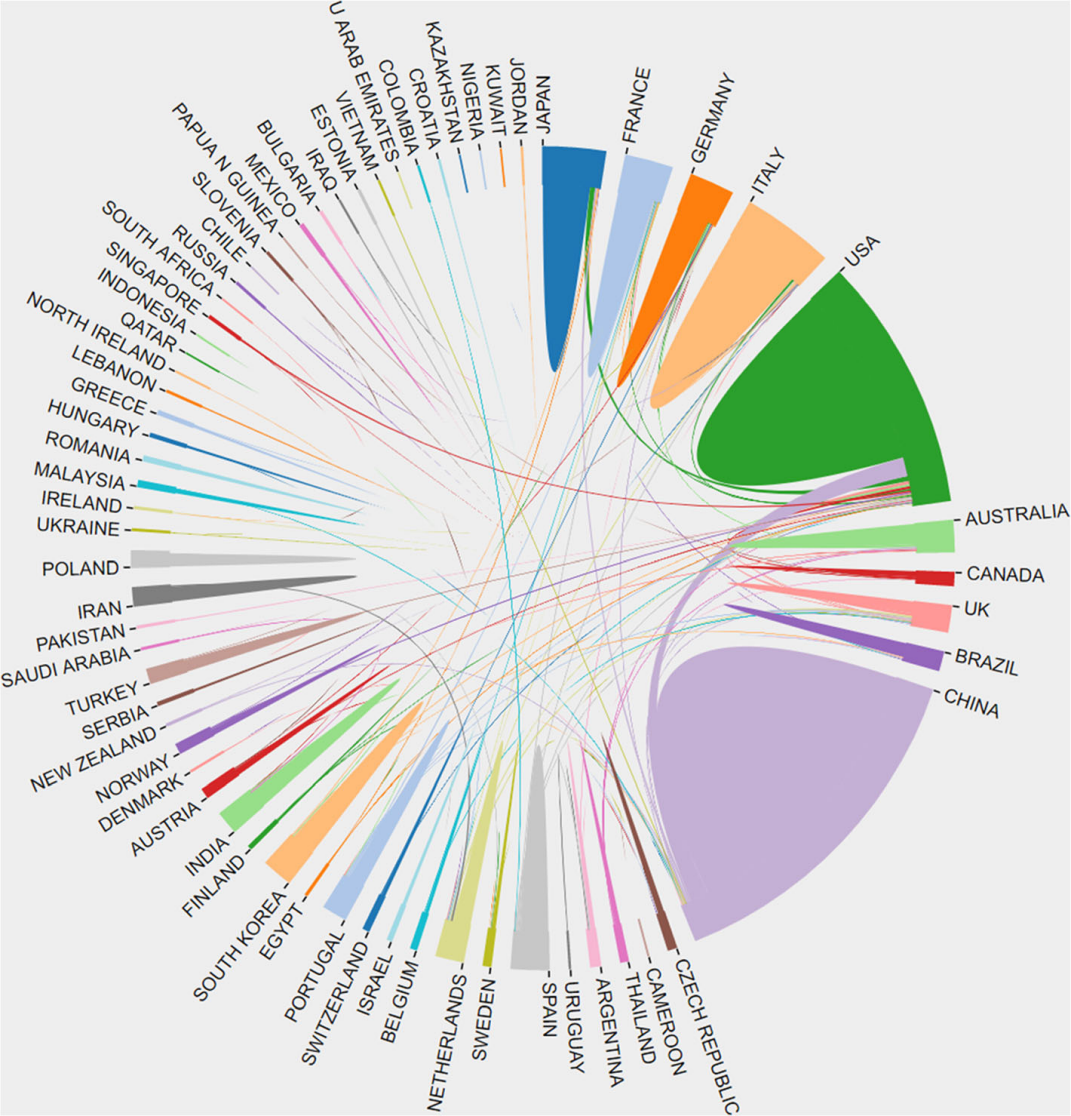
The cooperation between countries on osteosarcoma and cure

At least 64 different countries or regions participated in publishing studies on osteosarcoma over the past 10 years (Fig. 3). China (869) was the largest contributor, followed by the United States (520), Italy (172), Japan (137) and Britain (91). Centrality is an important index in evaluating the importance of nodes in a network in that the higher the centrality, the greater the effect of the nodes. We found that the influence of the United States is highly prominent with centrality = 0.44, followed by the United Kingdom with centrality = 0.26, which ranks 5th in the number of publications (Table 1). In terms of institutions (a total of 2501), Chinese universities in the top 10 have posted significant papers. In addition, Central South University and the University of Texas MD Anderson Cancer Center have the highest average citation rates of 3.25 and 2.87, respectively (Table 1). The centrality of most institutions is < 0.1, indicating a low level of influence and a lack of cooperation over the past decade. Figure 4 reveals that the cooperation between the United States and China was the closest, followed by the cooperation between the United States and the United Kingdom. The figure also suggests an absence of academic exchanges between countries with abundant publications and countries with weak publications.

**Table 1:** The top 10 countries and institutions contributing to publications on osteosarcoma and cure

Rank	Country	Articles	Centrality	Institutions	Articles	Total number of citations	Centrality	Average number of citations	Total number of first authors	Total number of first author citations	Average number of first author citations
1	China	869	0.11	Shanghai Jiao Tong Univ	100	233	0.12	2.33	43	84	1.95
2	USA	520	0.44	Jilin Univ	99	120	0.02	1.21	41	66	1.61
3	Italy	172	0.24	Chongqing Med Univ	86	148	0.01	1.72	21	27	1.29
4	Japan	137	0.05	Cent S Univ	84	273	0.02	3.25	36	131	3.64
5	UK	91	0.26	China Med Univ	83	163	0.06	1.96	21	26	1.24
6	Germany	90	0.19	Shandong Univ	71	174	0.09	2.45	31	55	1.77
7	India	89	0.05	Univ Texas MD Anderson Canc Ctr	69	198	0.06	2.87	22	59	2.68
8	France	75	0.06	Zhejiang Univ	54	119	0.03	2.2	29	78	2.69
9	Spain	70	0.05	Wuhan Univ	51	121	0.03	2.37	20	56	2.8
10	Australia	61	0.07	Ohio State Univ	51	106	0.17	2.08	12	20	1.67

### Journals publishing osteosarcoma and cure

The 2258 publications covered 772 journals, and the top 10 most popular journals published a total of 373 publications (16.52%). No significant difference was noted in the amount of papers issued by each journal, but the average citation rate of *BMC CANCER* was the highest at 3.26 (Table 2). Four journals have the characteristic of IF> 3: *International Journal of Molecular Sciences* (4.183), *Scientific Reports* (4.011), *International Journal of Oncology* (3.571) and *Oncology Reports* (3.041). The above four journals belong to Q1, Q1, Q1 and Q2, respectively, which are based on the JCR 2018 standard.

**Table 2:** The top 10 most active journals with publications on osteosarcoma and cure

Rank	Journal title	Articles (N)	Percentage (N/2258) %	IF (2018)	Quartile in category (2018)	H-index	Total number of citations	Average number of citations
1	ONCOTARGET	59	2.61	0	0	91	147	2.49
2	PLOS ONE	55	2.43	2.776	Q1	268	158	2.87
3	ONCOLOGY LETTERS	45	1.99	1.871	Q3	38	82	1.82
4	ONCOLOGY REPORTS	40	1.77	3.041	Q2	84	93	2.33
5	MOLECULAR MEDICINE REPORTS	37	1.64	1.851	Q3	43	60	1.62
6	INTERNATIONAL JOURNAL OF ONCOLOGY	28	1.24	3.571	Q1	111	58	2.07
7	ANTICANCER RESEARCH	28	1.24	1.935	Q3	110	38	1.36
8	BMC CANCER	27	1.20	2.933	Q2	111	88	3.26
9	SCIENTIFIC REPORTS	27	1.20	4.011	Q1	149	37	1.37
10	INTERNATIONAL JOURNAL OF MOLECULAR SCIENCES	27	1.20	4.183	Q1	114	24	0.89

### Contributions of authors to osteosarcoma and cure

**Fig. 5:**
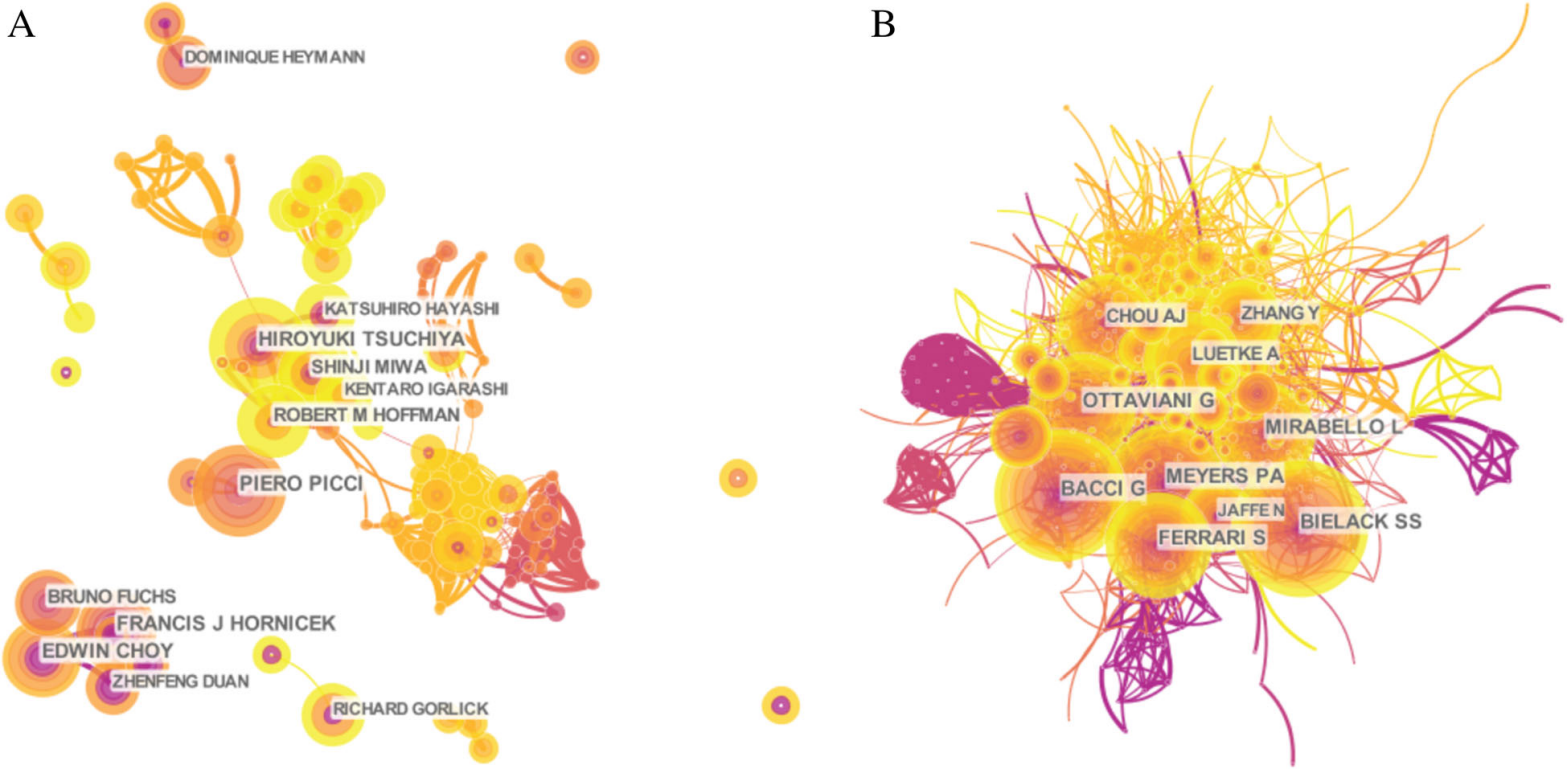
The distribution of authors engaged in osteosarcoma and cure. The network map of productive authors (**a**) and the network map of co-cited authors (**b**)

A total of 10,617 authors were filtered out in this study, and the top 10 authors who published the most papers are listed in Table 3. Two scholars, namely, Picci, P and Gorlick, R, had the highest average citation rates of 7.96 and 6.94, respectively, which demonstrated that they have made great achievements in the study of osteosarcoma and that their publications are of great academic value. The citation information of the authors and the co-cited authors were visualized by CiteSpace as two networks (Fig. 5). Bielack, SS (159) ranked first among the top ten co-citation authors, followed by Bacci, G (156) and Meyers, PA (137) (Table 3). It can be observed that these researchers have become influential experts in the field of osteosarcoma and have reported a substantial amount of research.

**Table 3:** Each of top 10 authors (most publications and co-cited) on osteosarcoma and cure

Rank	Author	Articles	Total number of citations	Average number of citations	First author counts	First author citation counts	Average First author citation counts	Corresponding author	Corresponding author citation counts	Co-cited author	Citation counts
1	Picci, P	28	223	7.96	1	15	15	1	15	Bielack, SS	159
2	Duan, ZF	24	80	3.33	3	23	7.67	18	58	Bacci, G	156
3	Hornicek, FJ	22	117	5.32	0	0	0	1	0	Meyers,PA	137
4	Tsuchiya, H	22	47	2.14	0	0	0	6	27	Ferrari,S	129
5	Choy, E	20	111	5.55	0	0	0	0	0	Mirabello,L	124
6	Heymann, D	20	77	3.85	0	0	0	8	47	Ottaviani,G	123
7	Gorlick, R	17	118	6.94	3	26	8.67	5	64	Chou,AJ	114
8	Hayashi, K	16	25	1.56	0	0	0	0	0	Luetke,A	114
9	Miwa, S	15	23	1.53	5	11	2.2	2	2	Zhang,Y	93
10	Fuchs, B	14	39	2.79	0	0	0	8	24	Jaffe,N	89

### Analysis of hotspots

**Fig. 6:**
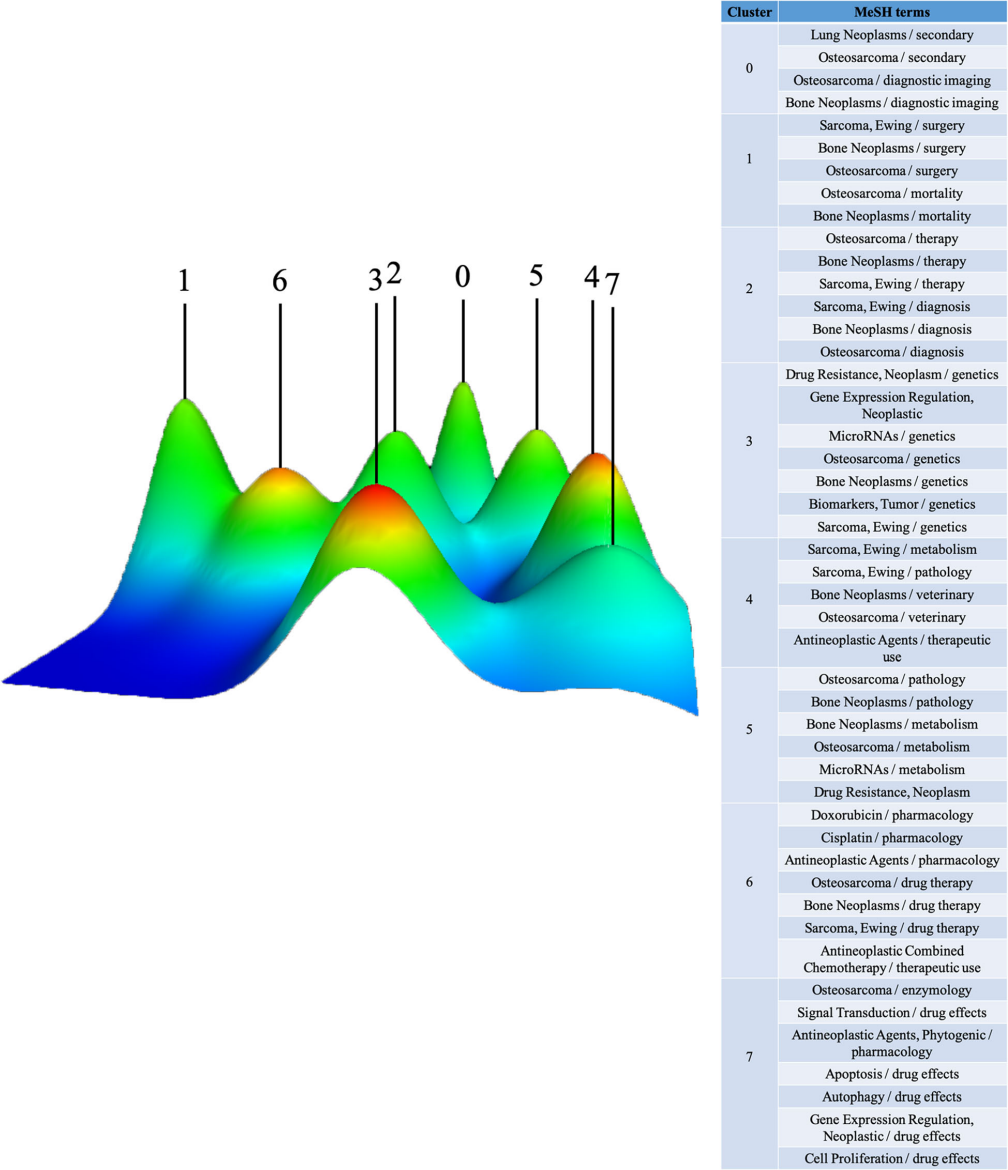
Mountain visualization of biclustering of highly frequent major MeSH terms and articles on osteosarcoma and cure. The volume of the peaks indicates the quantity of extremely frequent major MeSH terms/MeSH subheadings. The height and color of peaks are proportional to internal similarity and standard deviation (blue: high deviation; Red: low deviation.) Interval implies the relative similarity between them. At least 30 publications in each cluster and no triplet perks

**Fig. 7:**
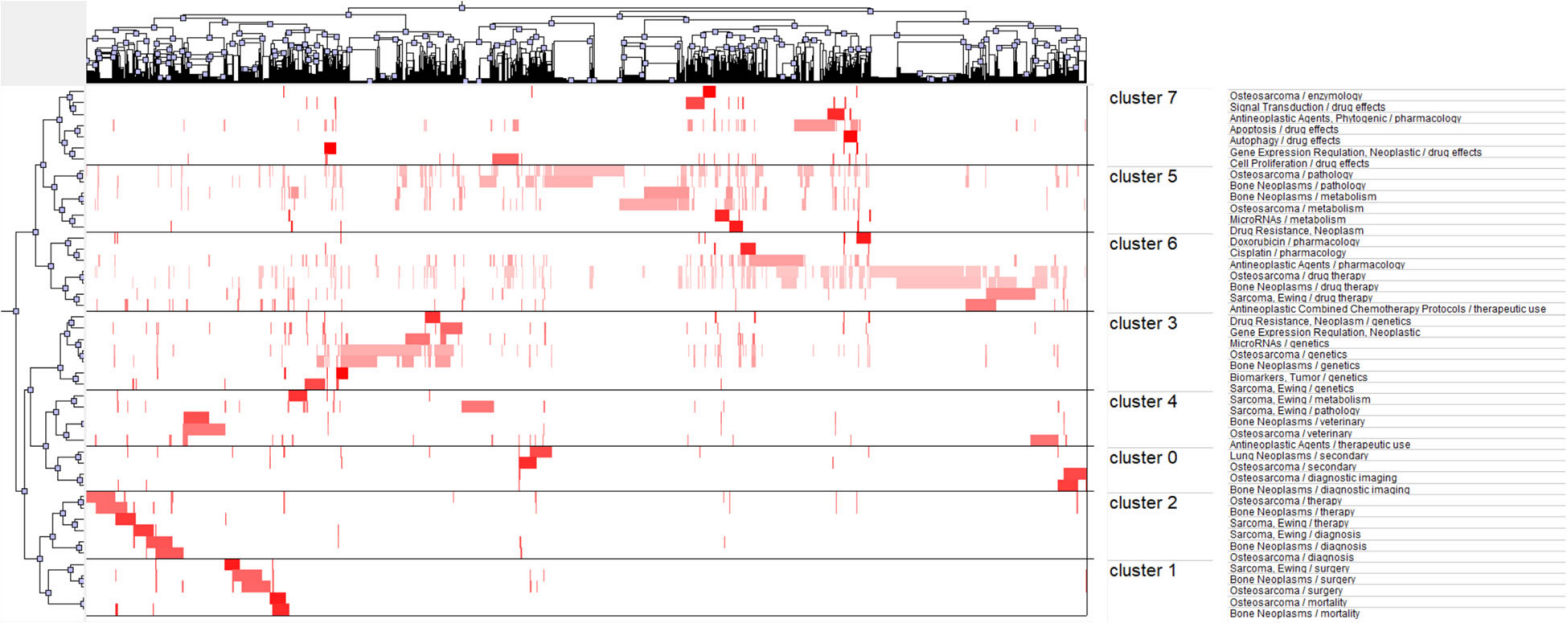
Visualized matrix of biclustering of highly frequent major MeSH terms and PubMed Unique Identifiers (PMIDs) of articles on osteosarcoma and cure. The cluster at the top indicates the literature, and the left represents extremely frequent major MeSH terms/MeSH subheadings. The color of the grid is relative to the frequency of it. (dark red is significant, white is close to non-significant)

In our study, we retrieved a total of 9084 major MeSH terms/MeSH subheadings with a cumulative frequency of 24,485.We defined terms that appear more than 50 times after evaluation by H index as extremely frequent terms, and forty-seven terms extracted from publications accounting for 37.61% (9209/24485) are shown in Table 4. The eight clusters (0–7) identified by bi-clustering were visualized in mountain form to indicate the quantity of extremely frequent major MeSH terms/MeSH subheadings and in matrix form to present the association between the source literature and MeSH terms/MeSH subheadings (Figs. 6 and 7). To facilitate similar row convergence in a single aggregation cluster, we reset the rows of the initial matrix through gCLUTO and divided each cluster with a black horizontal line (Table 5). Finally, we concluded on the following eight hot items with in-depth interpretation of the corresponding literatures:

**Table 4:** Highly frequent major MeSH terms from the included publications on osteosarcoma and cure (*n* = 24,485)

Rank	Major MeSH terms/ MeSH subheadings	Frequency	Proportion of frequency (%)	Cumulative percentage (%)
1	Osteosarcoma / drug therapy	984	4.0188	4.0188
2	Bone Neoplasms / drug therapy	824	3.3653	7.3841
3	Osteosarcoma / pathology	709	2.8957	10.2798
4	Bone Neoplasms / pathology	513	2.0952	12.3749
5	Osteosarcoma / genetics	505	2.0625	14.4374
6	Osteosarcoma / metabolism	457	1.8664	16.3039
7	Antineoplastic Agents / pharmacology	360	1.4703	17.7741
8	Bone Neoplasms / genetics	352	1.4376	19.2118
9	Bone Neoplasms / metabolism	306	1.2497	20.4615
10	Apoptosis / drug effects	292	1.1926	21.6541
11	Sarcoma, Ewing / drug therapy	246	1.0047	22.6588
12	Antineoplastic Combined Chemotherapy / therapeutic use	186	0.7596	23.4184
13	Osteosarcoma / veterinary	176	0.7188	24.1372
14	Sarcoma, Ewing / pathology	171	0.6984	24.8356
15	MicroRNAs / genetics	166	0.678	25.5136
16	Antineoplastic Agents / therapeutic use	165	0.6739	26.1875
17	Bone Neoplasms / therapy	150	0.6126	26.8001
18	Osteosarcoma / therapy	149	0.6085	27.4086
19	Gene Expression Regulation, Neoplastic	141	0.5759	27.9845
20	Cell Proliferation / drug effects	137	0.5595	28.544
21	Osteosarcoma / surgery	137	0.5595	29.1035
22	Bone Neoplasms / surgery	135	0.5514	29.6549
23	Osteosarcoma / diagnosis	116	0.4738	30.1287
24	Bone Neoplasms / diagnosis	111	0.4533	30.582
25	Bone Neoplasms / veterinary	105	0.4288	31.0108
26	Lung Neoplasms / secondary	99	0.4043	31.4152
27	Sarcoma, Ewing / genetics	98	0.4002	31.8154
28	Osteosarcoma / diagnostic imaging	98	0.4002	32.2156
29	Signal Transduction / drug effects	97	0.3962	32.6118
30	Bone Neoplasms / diagnostic imaging	87	0.3553	32.9671
31	Sarcoma, Ewing / diagnosis	85	0.3472	33.3143
32	Sarcoma, Ewing / therapy	82	0.3349	33.6492
33	Sarcoma, Ewing / metabolism	78	0.3186	33.9677
34	Drug Resistance, Neoplasm / genetics	77	0.3145	34.2822
35	Osteosarcoma / secondary	73	0.2981	34.5804
36	Antineoplastic Agents, Phytogenic / pharmacology	72	0.2941	34.8744
37	Cisplatin / pharmacology	72	0.2941	35.1685
38	Bone Neoplasms / mortality	71	0.29	35.4584
39	MicroRNAs / metabolism	68	0.2777	35.7362
40	Doxorubicin / pharmacology	62	0.2532	35.9894
41	Osteosarcoma / mortality	62	0.2532	36.2426
42	Drug Resistance, Neoplasm	62	0.2532	36.4958
43	Sarcoma, Ewing / surgery	62	0.2532	36.749
44	Autophagy / drug effects	54	0.2205	36.9696
45	Biomarkers, Tumor / genetics	53	0.2165	37.186
46	Osteosarcoma / enzymology	53	0.2165	37.4025
47	Gene Expression Regulation, Neoplastic / drug effects	50	0.2042	37.6067

**Table 5:** The representative completed clinical trials about osteosarcoma

No.	Study Title	Conditions	Interventions
1	Investigation of [6R] 5,10-methylenetetrahydrofolate as Rescue Therapy for Osteosarcoma Patients Treated with HDMTX	Osteosarcoma	Drug: Calcium Folinate and [6R] 5,10-methylenetetrahydrofolate
2	Phase II Study of Chemotherapy and Pamidronate for the Treatment of Newly Diagnosed Osteosarcoma	Osteosarcoma	Drug: Cisplatin, Doxorubicin and Methotrexate
3	Glembatumumab Vedotin in Treating Patients with Recurrent or Refractory Osteosarcoma	Recurrent Osteosarcoma	Drug: Glembatumumab Vedotin Other: Laboratory Biomarker Analysis And Pharmacological Study
4	Eribulin Mesylate in Treating Patients with Recurrent or Refractory Osteosarcoma	Recurrent Osteosarcoma	Drug: Eribulin Mesylate Other: Pharmacological Study
5	Chemotherapy for Patients with Osteosarcoma	Osteosarcoma	Drug: Pemetrexed
6	Inhalation SLIT Cisplatin (Liposomal) for the Treatment of Osteosarcoma Metastatic to the Lung	Osteosarcoma Metastatic	Drug: Cisplatin liposomal
7	Preventing Nephrotoxicity and Ototoxicity from Osteosarcoma Therapy	Osteosarcoma	Drug: Pantoprazole and High-dose methotrexate infusion duration
8	Inhaled Sargramostim in Treating Patients with First Pulmonary (Lung) Recurrence of Osteosarcoma	Metastatic Cancer Sarcoma	Biological: sargramostim Procedure: conventional surgery
9	Differentiation of Bone Sarcomas and Osteomyelitis with Ferumoxytol-Enhanced MRI	Bone Cancer Osteosarcoma	Drug: Feraheme Procedure: Magnetic Resonance Imaging (MRI) scan
10	Dacarbazine for Metastatic Soft Tissue and Bone Sarcoma	Sarcoma	Drug: Dacarbazine
11	A Study of Bevacizumab in Combination with Chemotherapy for Treatment of Osteosarcoma	Osteosarcoma	Biological: Bevacizumab Drug: Cisplatin, Doxorubicin, Methotrexate, Ifosfamide, etoposide Procedure: Surgery Radiation: Radiotherapy
12	A Phase II Study of Oral Cyclophosphamide and Sirolimus (OCR) in Advanced Sarcoma	Osteosarcoma	Drug: Cyclophosphamide and Sirolimus
13	Samarium Sm 153 and Stem Cell Transplant Followed by Radiation Therapy Patients with Osteosarcoma	Sarcoma	Biological: filgrastim Drug: ifosfamide Procedure: peripheral blood stem cell transplantation Radiation: Sm-EDTMP (low dose) and Sm-EDTMP (higher dose)
14	Gemcitabine and Docetaxel in Treating Patients with Recurrent Osteosarcoma (Closed to Accrual as of 12/21/06) or Ewing’s Sarcoma or Unresectable or Locally Recurrent Chondrosarcoma	Sarcoma	Biological: filgrastim and pegfilgrastim Drug: docetaxel and gemcitabine hydrochloride Genetic: microarray analysis Other: laboratory biomarker analysis and pharmacokinetic study
15	Temsirolimus and Cixutumumab in Treating Patients with Locally Advanced, Metastatic, or Recurrent Soft Tissue Sarcoma or Bone Sarcoma Metastatic Osteosarcoma	Recurrent Osteosarcoma	Biological: Cixutumumab Other: Laboratory Biomarker Analysis Drug: Temsirolimus
16	Therapeutic Angiotensin-(1–7) in Treating Patients with Metastatic Sarcoma That Cannot Be Removed by Surgery	Bone Cancer Metastatic Osteosarcoma	Drug: therapeutic angiotensin-(1–7) Other: laboratory biomarker analysis
17	Sorafenib in Treating Patients with Soft Tissue Sarcomas (Extremity Sarcoma Closed to Entry as of 5/30/07)	Metastatic Osteosarcoma Recurrent Osteosarcoma	Drug: sorafenib tosylate Procedure: therapeutic conventional surgery, computed tomography and dynamic contrast-enhanced magnetic resonance imaging Other: laboratory biomarker analysis and pharmacological study
18	Safety and Efficacy Study of Torisel and Liposomal Doxorubicin for Patients with Recurrent Sarcoma	Sarcoma	Drug: temsirolimus plus liposomal doxorubicin
19	A Study of Pemetrexed in Children with Recurrent Cancer	Osteosarcoma	Drug: pemetrexed
20	Vismodegib and Gamma-Secretase/Notch Signalling Pathway Inhibitor RO4929097 in Treating Patients with Advanced or Metastatic Sarcoma Metastatic Osteosarcoma	Recurrent Osteosarcoma	Drug: Gamma-Secretase Inhibitor RO4929097 and Vismodegib Other: Laboratory Biomarker Analysis and Pharmacological Study
21	Study to Find a Safe Dose and Show Early Clinical Activity of Weekly Nab-paclitaxel in Pediatric Patients with Recurrent/ Refractory Solid Tumors	Osteogenic Sarcoma	Drug: nab-paclitaxel
22	Cixutumumab and Temsirolimus in Treating Younger Patients with Recurrent or Refractory Sarcoma	Recurrent Osteosarcoma	Biological: Cixutumumab Other: Laboratory Biomarker Analysis Drug: Temsirolimus
23	Cixutumumab in Treating Patients with Relapsed or Refractory Solid Tumors	Recurrent Osteosarcoma	Biological: cixutumumab Other: laboratory biomarker analysis
24	Alisertib in Treating Young Patients with Recurrent or Refractory Solid Tumors or Leukemia	Recurrent Osteosarcoma	Drug: Alisertib Other: Laboratory Biomarker Analysis and Pharmacological Study
25	Depsipeptide (Romidepsin) in Treating Patients with Metastatic or Unresectable Soft Tissue Sarcoma	Adult Extraskeletal Osteosarcoma	Drug: romidepsin
26	Eurosarc Trial of Linsitinib in Advanced Ewing Sarcoma	Relapsed Ewing Sarcoma Refractory Ewing Sarcoma	Drug: Linsitinib
27	Olaparib in Adults with Recurrent/Metastatic Ewing’s Sarcoma	Ewing’s Sarcoma	Drug: Olaparib
28	Cytarabine in Treating Young Patients with Recurrent or Refractory Ewing’s Sarcoma	Sarcoma	Drug: cytarabine
29	Vinblastine, Celecoxib, and Combination Chemotherapy in Treating Patients with Newly-Diagnosed Metastatic Ewing’s Sarcoma Family of Tumors	Sarcoma	Drug: celecoxib, cyclophosphamide, doxorubicin hydrochloride, etoposide, ifosfamide, vinblastine sulfate, vincristine sulfate, MESNA and Filgrastim Procedure: conventional surgery Radiation: radiation therapy
30	Sunitinib in Treating Patients with Metastatic, Locally Advanced, or Locally Recurrent Sarcomas	Sarcoma	Drug: sunitinib malate
31	Vincristine Sulfate, Topotecan Hydrochloride, and Cyclophosphamide with or Without Bevacizumab in Treating Young Patients with Refractory or First Recurrent Extracranial Ewing Sarcoma	Ewing Sarcoma of Bone	Drug: topotecan hydrochloride, vincristine sulfate and cyclophosphamide Biological: bevacizumab
32	Cyclophosphamide, Topotecan, and Bevacizumab (CTB) in Patients with Relapsed/Refractory Ewing’s Sarcoma and Neuroblastoma	Neuroblastoma Sarcoma	Drug: Cyclophosphamide, Topotecan, and Bevacizumab
33	A Five-Tier, Open-Label Study of IMC-A12 in Advanced Sarcoma Ewing’s	Sarcoma	Biological: IMC-A12 (cixutumumab)
34	Trial of Dasatinib in Advanced Sarcomas	Sarcoma, Ewing’s	Drug: Dasatinib
35	Trabectedin in Treating Young Patients with Recurrent or Refractory Soft Tissue Sarcoma or Ewing’s Family of Tumors	Recurrent Ewing Sarcoma	Drug: trabectedin Other: pharmacological study
36	A Pilot Study of Autologous T-Cell Transplantation with Vaccine Driven Expansion of Anti-Tumor Effectors After Cytoreductive Therapy in Metastatic Pediatric Sarcomas	Ewing’s Sarcoma Rhabdomyosarcoma	Biological: therapeutic autologous dendritic cells Drug: indinavir sulfate Procedure: peripheral blood stem cell transplantation
37	Combination Chemotherapy and Peripheral Stem Cell Transplantation in Treating Patients with Sarcoma	Sarcoma	Biological: filgrastim Drug: cisplatin, doxorubicin hydrochloride, ifosfamide and melphalan Procedure: peripheral blood stem cell transplantation
38	Study Of CP-751,871 In Patients with Ewing’s Sarcoma Family of Tumors	Ewing’s Sarcoma	Drug: CP-751,871
39	Arsenic Trioxide in Treating Patients with Advanced Neuroblastoma or Other Childhood Solid Tumors	Sarcoma	Drug: arsenic trioxide
40	Plerixafor After Radiation Therapy and Temozolomide in Treating Patients with Newly Diagnosed High Grade Glioma	Adult Medulloblastoma	Radiation: radiation therapy Drug: temozolomide and plerixafor Other: laboratory biomarker analysis and pharmacological study
41	Therapy to Treat Ewing’s Sarcoma, Rhabdomyosarcoma or Neuroblastoma	Sarcoma	Drug: Tumor Purged/CD25 Depleted Lymphocytes and rhIL-7 Biological: Tumor Purged/CD25 Depleted Lymphocytes with Tumor Lysate/KLH Pulsed Dendritic Cell Vaccine and Tumor Lysate/KLH Pulsed Dendritic Cell Vaccine

New insights into the metastatic pattern of osteosarcoma (cluster 0),

Establishment of new methods for evaluating the sensitivity of chemotherapeutic drugs (cluster 1),

Alternatives to cytotoxic chemotherapy (cluster 2),

Prognostic markers (cluster 3),

Synergistic therapy of mesenchymal stem cells and drugs (cluster 4),

Reverse chemoresistance in osteosarcoma (cluster 5),

Drug discovery of Chinese herbal extracts to interfere with apoptosis of tumour cells (cluster 6),

Drug discovery of Chinese herbal extracts to interfere with autophagy of tumour cells (cluster 7).

## Discussion

Our statistical and quantitative analysis found a significant improvement in research on osteosarcoma and cure from 2010 to 2019, and an increasing number of orthopaedic and oncology experts focused their insights on this field. Although those studies have been highly extensive, they are relatively chaotic and lack hotspot analysis. Osteosarcoma metastasis, recurrence and multi-drug resistance (MDR) are the three major obstacles in the clinic, we discussed and explained the main 8 clusters obtained from co-word bi-clustering. In addition, we also collated 41 completed clinical trials in osteosarcoma that passed phase II (Table 5) and predicted future research trends by addressing these barriers.

Cluster 0 addresses the new insights on metastatic pattern. The survival rate of osteosarcoma is closely related to tumour metastasis in that 15–20% of patients were diagnosed with distant metastatic lesions at their first visit [14]. Previous studies have recognized that the lung is the most sensitive site, but the occurrence of abdominal metastasis reveals an unusual pattern in which osteosarcoma is more prone to occur in soft tissue [15]. Changes in the tumour microenvironment and extracellular matrix composition seriously affect osteosarcoma metastasis, e.g., the “seed and soil hypothesis”. The rich bone microenvironment constitutes a fertile “soil” that favours the growth of both primary and metastatic tumoural “seeds”, and the host “soil” is transferred to undergo biological changes in advance to adapt to the invasion of “seeds” [16]. Clinicians should be aware of the development of metastatic patterns in patients with osteosarcoma, and the exploration of microenvironmental tumour genetic disorders between primary occurrence and metastasis is the subject of future basic research.

Cluster 1 investigates new methods for evaluating the sensitivity of chemotherapeutic drugs. The sensitivity of osteosarcoma to chemotherapeutics drugs plays a critical role in the choice of operation and in tumour recurrence. Current standard treatments for osteosarcoma include neoadjuvant multidrug chemotherapy, growth factor support, surgery (amputation or limb salvage), and radiotherapy [17]. It is known that limb salvage surgery still has many complications, such as local recurrence, and amputees are more likely to suffer from metastasis and a lower survival rate and that all of those complications are attributed to insufficient chemotherapy [18]. Hence, a new reliable method for determining the effectiveness of chemotherapeutic drugs prior to surgery is expected to aid surgeons in choosing surgical tactics, such as radiological functional imaging (dynamic contrast enhanced magnetic resonance imaging, magnetic resonance diffusion weighted and deoxyglucose positron emission tomography). These approaches were applied to clinical studies (Table 5; No. 9, 17).

Cluster 2 involves alternatives to cytotoxic chemotherapy. Currently, no effective alternative therapy is available to treat osteosarcoma after failure of traditional chemotherapy and surgery. Based on the literature in the past 10 years, we found that researchers at home and abroad are actively exploring strategies to replace systemic chemotherapy for osteosarcoma: (1) immunotherapy, including active (cancer vaccine, adoptive T-cell transfer (Table 5; No. 36), checkpoint inhibitor (programmed cell death-ligand 1, T-cell immunoglobulin, mucin-domain containing-3, Indoleamine 2,3-di-oxygenase) and cytokines) and passive (monoclonal antibodies and adjuvants (Table 5; No. 11, 15, 22, 23)) approaches [19]; (2) local chemotherapy in which implantable drug delivery systems with anticancer drugs and bone substitutes as carriers can protect drugs from rapid metabolism in circulation and ensure the objective therapeutic effect of low dose therapies such as cyclodextrins, poly-lactic-co-glycolic acid and cyclodextrins [20], and nanoparticle albumin binding paclitaxel could increase the drug concentration in tumour cells (Table 5; No. 21); (3) radiotherapy, including stereotactic radiosurgery, carbon-ion radiotherapy, etc. [21, 22]; (4) genetic engineering, e.g., biological genetic engineering-edited miRNAs that target RNA molecules [23]; (5) stem cell therapy (Table 5; No. 13, 37); and (6) novel alternative therapeutic drugs originating from Chinese herbal extracts. These two points are discussed in detail later on. In terms of the future, with the rapid development of biotechnology, current research is focusing on alternative methods of impairing osteosarcoma outside of cytotoxic chemotherapy.

Cluster 3 explores new prognostic markers for osteosarcoma. The urgent need exists for early diagnosis, and it is beneficial to adopt a better therapeutic schedule. The existing clinical trials focus on that topic (Table 5). Studies have shown that a variety of miRNAs in osteosarcoma tissues or blood samples have been altered and are closely associated with poor prognosis of either deaths or events [24], such as high expression of miRNA 210, 17–92 cluster, 128, 9, 214, 542-5p, 130b, 130a, and 199b-5p and low expression of miRNA 132, 145, 382, 133a, 26a, 340, 20a, 92a, 143, 451, 144, 22, 195, 124, 449a, 99a, and 224 [25]. In addition, large-scale prospective studies are expected to elucidate the prognostic role of miRNAs in osteosarcoma, and the role of these miRNAs in assessing tumour progression and therapeutic responses will depend on additional clinical trials.

Cluster 4 is related to synergistic therapy of mesenchymal stem cells (MSCs) and drugs. Tumour cells in osteosarcoma are similar to osteoblasts in that they present osteoblastic differentiation and produce malignant osteoid, not only in osteoblastic areas but also in chondroblast or fibroblast areas, which suggests that osteosarcoma cells might originate from MSCs. We know that MSCs are highly prone to tumour stroma and can promote MDR by increasing the expression of multi-drug resistance genes or by paracrine pathways (STAT3, IL-6, IL-8, etc.) [26]. By taking advantage of this knowledge, we can load MSCs with therapeutic drugs to exert their anticancer capacity more effectively. It has been reported that rhBMP-2 and MSCs combined with conventional antitumour therapy might be an efficient therapeutic strategy against osteosarcoma [27]. However, conventional chemicals as the carriers in MSCs might disrupt the normal function of MSCs and cause drug delivery failure. Duchi found that photodynamic therapy, in which MSCs are stimulated by light activation to release toxic drugs that kill the surrounding tumour cells and induce their own death, might be a good option [28]. Furthermore, MSCs transfected with adenovirus carrying the osteoprotegerin gene inhibited osteosarcoma growth and bone destruction [29]. In addition to cytotoxic drugs and viral vectors, anti-angiogenic agents and immunostimulants can also be delivered to tumour lesions by MSCs [30]. Existing clinical trials explored stem cell transplantation to treat sarcomas (Table 5, No. 13, 37). In the next few decades, MSCs are expected to certainly display their advantages in carrying targeted drugs or antitumour genes for osteosarcoma treatment and are an exploration direction for researchers.

Cluster 5 focuses on reverse chemoresistance. Studies over the past 10 years reflect that chemotherapy for osteosarcoma has entered a bottleneck period. Even with increasing the dose, the types of drugs combined with chemotherapy and alteration of the delivery pathway still cannot improve the 5-year survival rate. Although MAP (methotrexate, doxorubicin and cisplatin) has become the preferred clinical treatment regimen, the problem of MDR still exists due to the following mechanisms: the reduction of effective intracellular accumulation, the abnormal content or activity of drug metabolism enzymes, and the genomic complexity and tumour heterogeneity of osteosarcoma [31]. Enhanced cell detoxification, DNA damage repair, apoptosis inhibition and autophagy-related chemoresistance also contributed to the emergence of osteosarcoma MDR, which is the main reason for the low survival rate. Targeted and precise individual therapy is the key step in overcoming MDR [32]. Exploration of MDR-related molecules such as proteins, enzymes, miRNA, lncRNA, circular RNA, cholesterol, folic acid, etc., and development of those molecules to biological targets for reversal of MDR have become a current focus [33–39]. For example, clinical trials with folic acid and enzymes have been conducted, as shown in Table 5 (No. 1, 5, 25). It is worth noting that invariant NKT cells, a lymphocyte lineage with features of both T and NK cells, can significantly enhance the cytotoxicity induced by cisplatin, doxorubicin and methotrexate on osteosarcoma cells [40]. T-cell transplantation has advantages in metastatic paediatric sarcomas treatment (Table 5; No. 36). Efforts to actively reveal additional potential molecular mechanisms of MDR, develop novel molecular-targeted drugs depending on the related signalling pathways, and conduct drug evaluation experiments are expected to remain a hot topic from past and present to the future.

Cluster 6 focuses on drug discovery of Chinese herbal extracts that interfere with apoptosis of tumour cells. As described in the previous paragraph, chemoresistance of osteosarcoma is the most prominent clinical dilemma. Recently, traditional Chinese medicines have become increasingly vital in treatment of tumours and are a good source of new anticancer drugs due to advantages of fewer side effects and the absence of chemoresistance [41]. For instance, eribulin mesylatein has been applied to clinical trial (Table 5’ No. 4). Tracing back through the past decade of traditional Chinese medicine studies on osteosarcoma, we found that many of them focused on interfering with the apoptosis of tumour cells, which was considered as the main way to eradicate cancer, and several natural ingredients with potential clinical applications have been explored, such as celastrol, honokiol, berberine, chamaejas-mine and artemisinin [42–46]. In addition, natural compounds responded well to cell cycle arrest and apoptosis with increased reactive oxygen species and could improve the 5-year survival rate. Therefore, it is necessary to find additional Chinese herbal extracts that regulate the oxidative stress level to intervene in the apoptosis of osteosarcoma cells.

Cluster 7 addresses drug discovery of Chinese herbal extracts that interfere with autophagy of tumour cells. It is well known that autophagy-induced cell death is another important indicator for evaluating anticancer drugs in addition to apoptosis. In fact, autophagy can promote cell survival and lead to cell death [47, 48]. This critical process of autophagy is deregulated in osteosarcoma, but it can be induced [49]. Consistently, regulation of tumour cell autophagy by natural Chinese herbal medicine ingredients is also a current hot topic with substances such as riccardin and celastrol [45, 50]. Notably, autophagy is still highly related to MDR and prognosis of osteosarcoma, which further solidifies the discoverability among osteosarcoma chemoresistance, traditional Chinese medicine extracts and autophagy [51]. The occurrence of osteosarcoma does not follow a simple pattern. The complexity of the genome promotes the drug resistance of osteosarcoma and supplies infinite possibilities for new medicine. Phenotypic screening of various active ingredients in natural Chinese herbal medicine is expected to promote the development of new compounds based on apoptosis and autophagy.

Nevertheless, we were aware of several potential limitations of this study. First, those novel and less-focused MeSH terms may not be involved. Second, the number of related papers on osteosarcoma and cure may increase rapidly, that the constant updating of the database may lead to a difference between the bibliographic analysis data and the actual research progress. Finally, the error of the database itself may lead to a deviation of the result, such as Web of Science may mislabel document types, so that the dataset may contain papers that should be filtered out and miss papers that should be included.

## Conclusions

In our study, we collected new ideas on the metastasis pattern of osteosarcoma, emphasizing the abnormality of the tumour microenvironment genetics. The existence and prevalence of multi-drug resistance make the emergence of new alternative tactics and multi-dimensional evaluation systems to replace systemic chemotherapy a crucial effort. Ongoing clinical trials and a 10-year literature search also corroborated that the development of novel drugs to overcome chemoresistance is a top priority, and extracts from traditional Chinese medicine offer new resources. Overall, we believe that this article is of guiding significance for osteosarcoma and that the above hotspots might contribute to a breakthrough in the future.

## Data Availability

The datasets supporting the conclusions of this article are available from the corresponding authors upon reasonable request.

## Abbreviations

BICOMB: Bibliographic item co-occurrence matrix builder
MeSH: Medical Subject Headings
MDR: multi-drug resistance
MSCs: mesenchymal stem cells
